# Schur convexity of the generalized geometric Bonferroni mean and the relevant inequalities

**DOI:** 10.1186/s13660-017-1605-7

**Published:** 2018-01-08

**Authors:** Huan-Nan Shi, Shan-He Wu

**Affiliations:** 1 Department of Mathematics, Longyan University, Longyan, Fujian 364012 P.R. China; 2 Department of Electronic Information, Beijing Union University, Beijing, 100011 P.R. China

**Keywords:** 26E60, 26B25, geometric Bonferroni mean, Schur’s condition, majorization relationship, inequality

## Abstract

In this paper, we discuss the Schur convexity, Schur geometric convexity and Schur harmonic convexity of the generalized geometric Bonferroni mean. Some inequalities related to the generalized geometric Bonferroni mean are established to illustrate the applications of the obtained results.

## Introduction

The Schur convexity of functions relating to special means is a very significant research subject and has attracted the interest of many mathematicians. There are numerous articles written on this topic in recent years; see [1, 2] and the references therein. As supplements to the Schur convexity of functions, the Schur geometrically convex functions and Schur harmonically convex functions were investigated by Zhang and Yang [3], Chu, Zhang and Wang [4], Chu and Xia [5], Chu, Wang and Zhang [6], Shi and Zhang [7, 8], Meng, Chu and Tang [9], Zheng, Zhang and Zhang [10]. These properties of functions have been found to be useful in discovering and proving the inequalities for special means (see [11–14]).

Recently, it has come to our attention that a type of means which is symmetrical on *n* variables $x_{1}, x_{2}, \ldots , x_{n}$ and involves two parameters, it was initially proposed by Bonferroni [15], as follows:

1 $$ B^{p,q}(\boldsymbol {x})= \Biggl( \frac{1}{n(n-1)}\sum ^{n}_{i,j=1,i\neq j} x^{p} _{i} x^{q}_{j} \Biggr) ^{\frac{1}{p+q}}, $$

where $\boldsymbol {x}=(x_{1},x_{2},\ldots,x_{n})$, $x_{i}\geq 0$, $i=1,2,\ldots,n$, $p,q \geq 0$ and $p+q\neq 0$.

$B^{p,q}(\boldsymbol {x})$ is called the Bonferroni mean. It has important application in multi criteria decision-making (see [16–21]).

Beliakov, James and Mordelová *et al.* [22] generalized the Bonferroni mean by introducing three parameters *p*, *q*, *r*, *i.e.*,

2 $$ B^{p,q,r}(\boldsymbol {x})= \Biggl( \frac{1}{n(n-1)(n-2)}\sum ^{n}_{i,j.k=1,i \neq j\neq k} x^{p}_{i} x^{q}_{j}x^{r}_{k} \Biggr) ^{\frac{1}{p+q+r}}, $$

where $\boldsymbol {x}=(x_{1},x_{2},\ldots,x_{n})$, $x_{i}\geq 0$, $i=1,2,\ldots,n$, $p,q,r \geq 0$ and $p+q+r \neq 0$.

Motivated by the Bonferroni mean $B^{p,q}(\boldsymbol {x})$ and the geometric mean $G(\boldsymbol {x})=\prod^{n}_{i=1}(x_{i})^{\frac{1}{n}}$, Xia, Xu and Zhu [23] introduced a new mean which is called the geometric Bonferroni mean, as follows:

3 $$ \operatorname {GB}^{p,q}(\boldsymbol {x})=\frac{1}{p+q}\prod^{n}_{i,j=1,i\neq j}(p x_{i}+q x _{j})^{\frac{1}{n(n-1)}}, $$

where $\boldsymbol {x}=(x_{1},x_{2},\ldots,x_{n})$, $x_{i} > 0$, $i=1,2,\ldots,n$, $p,q \geq 0$ and $p+q\neq 0$.

An extension of the geometric Bonferroni mean was given by Park and Kim in [19], which is called the generalized geometric Bonferroni mean, *i.e.*,

4 $$ \operatorname {GB}^{p,q,r}(\boldsymbol {x})=\frac{1}{p+q+r}\prod^{n}_{i,j,k=1,i\neq j\neq k}(p x_{i}+q x_{j}+r x_{k})^{\frac{1}{n(n-1)(n-2)}}, $$

where $\boldsymbol {x}=(x_{1},x_{2},\ldots,x_{n})$, $x_{i}> 0$, $i=1,2,\ldots,n$, $p,q,r \geq 0$ and $p+q+r \neq 0$.

### Remark 1

For $r=0$, it is easy to observe that

$$\begin{aligned} \operatorname {GB}^{p,q,0}(\boldsymbol {x})&=\frac{1}{p+q+0}\prod ^{n}_{i,j=1,i\neq j} \Biggl[\prod^{n}_{k=1,i\neq j\neq k}(p x_{i}+q x_{j}+0\times x_{k}) \Biggr]^{ \frac{1}{n(n-1)(n-2)}} \\ & =\frac{1}{p+q}\prod^{n}_{i,j=1,i\neq j} \bigl[(p x_{i}+q x_{j})^{(n-2)} \bigr]^{\frac{1}{n(n-1)(n-2)}} \\ & =\frac{1}{p+q}\prod^{n}_{i,j=1,i\neq j}(p x_{i}+q x_{j})^{ \frac{1}{n(n-1)}} \\ & =\operatorname {GB}^{p,q}(\boldsymbol {x}). \end{aligned}$$

### Remark 2

If $q = 0$, $r=0$, then the generalized geometric Bonferroni mean reduces to the geometric mean, *i.e.*,

$$ \operatorname {GB}^{p,0,0}(\boldsymbol {x})=\operatorname {GB}^{p,0}(\boldsymbol {x})=\frac{1}{p}\prod ^{n}_{i,j=1,i \neq j}(p x_{i})^{\frac{1}{n(n-1)}}= \prod^{n}_{i=1}(x_{i})^{ \frac{1}{n}}=G( \boldsymbol {x}). $$

### Remark 3

If $\boldsymbol {x}=(x,x,\ldots,x)$, then

$$ \operatorname {GB}^{p,q,r}(\boldsymbol {x})=\operatorname {GB}^{p,q,r}(x,x,\ldots,x)=x. $$

For convenience, throughout the paper $\mathbb{R}$ denotes the set of real numbers, $\boldsymbol {x} = (x_{1}, x_{2}, \ldots,x_{n} )$ denotes *n*-tuple (*n*-dimensional real vectors), the set of vectors can be written as

$$\begin{aligned}& \mathbb{R}^{n} = \bigl\{ {\boldsymbol {x}=(x_{1}, x_{2}, \ldots,x_{n} ): x _{i} \in \mathbb{R}, i = 1,2,\ldots,n} \bigr\} , \\& \mathbb{R}^{n}_{+}= \bigl\{ \boldsymbol {x}=(x_{1}, x_{2},\ldots,x_{n}): x_{i} \geq 0, i=1,2,\ldots,n \bigr\} , \\& \mathbb{R}^{n}_{++}= \bigl\{ \boldsymbol {x}=(x_{1}, x_{2},\ldots,x_{n}): x _{i}>0, i=1,2,\ldots,n \bigr\} . \end{aligned}$$

In a recent paper [24], Shi and Wu investigated the Schur *m*-power convexity of the geometric Bonferroni mean $\operatorname {GB}^{p,q}(\boldsymbol {x})$. The definition of Schur *m*-power convex function is as follows:

Let $f:\mathbb{R}_{++}\to \mathbb{R}$ be a function defined by

$$ f(x)= \textstyle\begin{cases} \frac{x^{m}-1}{m}, & m\ne 0, \\ \ln x, & m=0. \end{cases} $$

Then a function $\varphi :\Omega \subset \mathbb{R}_{++}^{n}\to \mathbb{R}$ is said to be Schur *m*-power convex on Ω if

$$ \bigl(f(x_{1}), f(x_{2}),\ldots,f(x_{n}) \bigr) \prec \bigl(f(y_{1}), f(y_{2}),\ldots,f(y_{n}) \bigr) $$

for all $(x_{1},x_{2},\ldots,x_{n})\in \Omega $ and $(y_{1},y_{2},\ldots,y_{n})\in \Omega $ implies $\phi (x)\le \phi (y)$.

If −*φ* is Schur *m*-power convex, then we say that *φ* is Schur *m*-power concave.

Shi and Wu [24] obtained the following result.

### Proposition 1

*For fixed positive real numbers* *p*, *q*, (i)* if* $m<0 $ *or* $m=0 $, *then* $\operatorname {GB}^{p,q}(\boldsymbol {x})$ *is Schur* *m*-*power convex on* $\mathbb{R}^{n}_{++}$; (ii)* if* $m=1 $ *or* $m\geq 2 $, *then* $\operatorname {GB}^{p,q}(\boldsymbol {x})$ *is Schur* *m*-*power concave on* $\mathbb{R}^{n}_{++}$.

In this paper we discuss the Schur convexity, Schur geometric convexity and Schur harmonic convexity of the generalized geometric Bonferroni mean $\operatorname {GB}^{p,q,r}(\boldsymbol {x})$. Our main results are as follows.

### Theorem 1

*For fixed non*-*negative real numbers* *p*, *q*, *r* *with* $p+q+r \neq 0$, *if* $\boldsymbol {x}=(x_{1},x_{2},\ldots,x_{n})$, $n\geq 3 $, *then* $\operatorname {GB}^{p,q,r}(\boldsymbol {x})$ *is Schur concave*, *Schur geometric convex and Schur harmonic convex on* $\mathbb{R}^{n}_{++}$.

### Corollary 1

*For fixed non*-*negative real numbers* *p*, *q* *with* $p+q\neq 0$, *if* $\boldsymbol {x}=(x_{1},x_{2},\ldots,x_{n})$, $n\geq 3 $, *then* $\operatorname {GB}^{p,q}(\boldsymbol {x})$ *is Schur concave*, *Schur geometric convex and Schur harmonic convex on* $\mathbb{R}^{n}_{++}$.

## Preliminaries

We introduce some definitions, lemmas and propositions, which will be used in the proofs of the main results in subsequent sections.

### Definition 1

(see [1])

Let $\boldsymbol {x} = ( x_{1},x_{2},\ldots, x_{n })$ and $\boldsymbol {y} = ( y_{1},y_{2},\ldots, y_{n }) \in \mathbb{R}^{n}$.

(i) ***x*** is said to be majorized by ***y*** (in symbols $\boldsymbol {x} \prec \boldsymbol {y}$) if $\sum_{i = 1}^{k} x_{[i]} \le \sum_{i = 1} ^{k} y_{[i]}$ for $k = 1,2,\ldots,n - 1$ and $\sum_{i = 1}^{n} x_{i} = \sum_{i = 1}^{n} y_{i}$, where $x_{[1]}\ge x_{[2]}\ge \cdots \ge x _{[n]}$ and $y_{[1]}\ge y_{[2]}\ge \cdots \ge y_{[n]}$ are rearrangements of ***x*** and ***y*** in a descending order.
(ii) Let $\Omega \subset \mathbb{R}^{n}$,the function *φ*: $\Omega \to \mathbb{R}$ is said to be Schur convex on Ω if $\boldsymbol {x} \prec \boldsymbol {y}$ on Ω implies $\varphi ( \boldsymbol {x} ) \le \varphi ( \boldsymbol {y} ) $. *φ* is said to be Schur concave function on Ω if and only if −*φ* is Schur convex function on Ω.

### Definition 2

(see [1])

Let $\boldsymbol {x} = ( x_{1},x_{2},\ldots, x_{n })$ and $\boldsymbol {y} = ( y_{1},y_{2},\ldots, y_{n }) \in \mathbb{R}^{n}$. $\Omega \subset \mathbb{R}^{n}$ is said to be a convex set if $\boldsymbol {x},\boldsymbol {y}\in \Omega $ and $0\leq \alpha \leq 1$ imply

$$ \alpha \boldsymbol {x}+(1-\alpha )\boldsymbol {y}= \bigl( \alpha x_{1}+(1-\alpha )y_{1} , \alpha x_{2}+(1-\alpha )y_{2},\ldots, \alpha x_{n}+(1-\alpha )y_{n} \bigr) \in \Omega . $$

### Definition 3

(see [1])

(i) A set $\Omega \subset \mathbb{R}^{n}$ is called symmetric, if $\boldsymbol {x}\in \Omega $ implies $\boldsymbol {x}P \in \Omega $ for every $n\times n$ permutation matrix *P*.

(ii) A function $\varphi : \Omega \to \mathbb{R}$ is called symmetric if for every permutation matrix *P* and $\varphi (\boldsymbol {x}P) = \varphi (\boldsymbol {x})$ for all $\boldsymbol {x} \in \Omega $.

The following proposition is called Schur’s condition. It provides an approach for testing whether a vector valued function is Schur convex or not.

### Proposition 2

(see [1])

*Let* $\Omega \subset \mathbb{R} ^{n} $ *be symmetric and have a nonempty interior convex set*. $\Omega^{0}$ *is the interior of *Ω. $\varphi :\Omega \to \mathbb{R} $ *is continuous on* Ω *and differentiable in* $\Omega^{0}$. *Then* *φ* *is the Schur convex function* (*Schur concave function*) *if and only if* *φ* *is symmetric on* Ω *and*

5 $$ ( x_{1} - x_{2} ) \biggl[ \frac{\partial \varphi ( \boldsymbol{x})}{\partial x_{1}} - \frac{\partial \varphi ( \boldsymbol{x})}{\partial x_{2} } \biggr] \ge 0\quad (\leq 0) $$

*holds for any* $\boldsymbol {x} \in \Omega^{0} $.

### Definition 4

(see [25])

Let $\boldsymbol {x} = ( x_{1}, x_{2},\ldots, x_{n })$ and $\boldsymbol {y} = ( y_{1},y _{2},\ldots, y_{n }) \in \mathbb{R}_{+}^{n}$.

(i) $\Omega \subset \mathbb{R}_{+} ^{n}$ is called a geometrically convex set if $(x_{1}^{\alpha }y_{1}^{\beta }, x_{2} ^{\alpha }y_{2}^{\beta },\ldots,x_{n}^{\alpha }y_{n}^{\beta }) \in \Omega $ for all ***x***, $\boldsymbol {y} \in \Omega $ and *α*, $\beta \in [0, 1]$ such that $\alpha +\beta =1$.
(ii) Let $\Omega \subset \mathbb{R}_{+} ^{n}$. The function *φ*: $\Omega \to \mathbb{R}_{+}$ is said to be Schur geometrically convex function on Ω if $( \log x_{1},\log x _{2},\ldots, \log x_{n }) \prec ( \log y_{1}, \log y_{2},\ldots, \log y_{n})$ on Ω implies $\varphi ( \boldsymbol {x} ) \le \varphi ( \boldsymbol {y} ) $. The function *φ* is said to be a Schur geometrically concave function on Ω if and only if −*φ* is Schur geometrically convex function.

### Proposition 3

(see [25])

*Let* $\Omega \subset \mathbb{R}_{+} ^{n} $ *be a symmetric and geometrically convex set with a nonempty interior* $\Omega^{0}$. *Let* $\varphi :\Omega \to \mathbb{R}_{+}$ *be continuous on* Ω *and differentiable in* $\Omega^{0}$. *If* *φ* *is symmetric on* Ω *and*

6 $$ ( {\log x_{1} -\log x_{2} } ) \biggl[ {x_{1} \frac{\partial \varphi (\boldsymbol {x})}{\partial x_{1} } - x_{2} \frac{\partial \varphi (\boldsymbol {x})}{ \partial x_{2} }} \biggr] \ge 0 \quad ( \leq 0) $$

*holds for any* $\boldsymbol {x}\in \Omega^{0} $, *then* *φ* *is a Schur geometrically convex* (*Schur geometrically concave*) *function*.

### Definition 5

(see [26])

Let $\Omega \subset \mathbb{R}_{+}^{n}$.

(i) A set Ω is said to be harmonically convex if $\frac{\boldsymbol{xy}}{\lambda {\boldsymbol{x}}+(1-\lambda ){\boldsymbol{y}}} \in \Omega $ for every ${\boldsymbol{x},\boldsymbol{y}}\in \Omega $ and $\lambda \in [0,1]$, where $\boldsymbol{xy}=(x_{1}y_{1},x_{2}y_{2},\ldots, x_{n}y_{n})$ and
  $$\frac{1}{\boldsymbol{\lambda x+(1-\lambda )y}} = \biggl( \frac{1}{\lambda x_{1}+(1-\lambda )y_{1}}, \frac{1}{\lambda x_{2}+(1- \lambda )y_{2}},\ldots, \frac{1}{\lambda x_{n}+(1-\lambda )y_{n}} \biggr). $$
(ii) A function $\varphi :\Omega \to \mathbb{R}_{+}$ is said to be Schur harmonically convex on Ω if $\frac{1}{\boldsymbol{x}} \prec \frac{1}{\boldsymbol{y}}$ implies $\varphi ({\boldsymbol{x}}) \le \varphi ({\boldsymbol{y}})$. A function *φ* is said to be a Schur harmonically concave function on Ω if and only if −*φ* is a Schur harmonically convex function.

### Proposition 4

(see [26])

*Let* $\Omega \subset \mathbb{R}_{+}^{n}$ *be a symmetric and harmonically convex set with inner points*, *and let* $\varphi :\Omega \to \mathbb{R}_{+}$ *be a continuously symmetric function which is differentiable on* $\Omega^{0}$. *Then* *φ* *is Schur harmonically convex* (*Schur harmonically concave*) *on* Ω *if and only if*

7 $$ (x_{1}-x_{2}) \biggl[ x_{1}^{2} \frac{\partial \varphi ({\boldsymbol{x}})}{ \partial x_{1}} -x_{2}^{2} \frac{\partial \varphi ({\boldsymbol{x}})}{ \partial x_{2}} \biggr] \ge 0\quad (\leq 0) $$

*holds for any* $\boldsymbol {x} \in \Omega^{0} $.

### Remark 4

Propositions 3 and 4 provide analogous Schur’s conditions for determining Schur geometrically convex functions and Schur harmonically convex functions, respectively.

### Lemma 1

(see [1])

*Let* $\boldsymbol {x}=( x_{1}, x_{2},\ldots, x_{n} )\in \mathbb{R}^{n}_{+} $ *and* $A_{n}(\boldsymbol {x})= \frac{1}{n}\sum_{i=1}^{n}x_{i}$. *Then*

8 $$ \bigl( \underbrace{ A_{n}(\boldsymbol {x}), A_{n}(\boldsymbol {x}),\ldots, A_{n}(\boldsymbol {x})}_{n} \bigr) \prec (x_{1}, x_{2},\ldots, x_{n}). $$

### Lemma 2

(see [1])

*If* $x_{i}>0$, $i=1,2,\ldots,n$, *then*, *for any non*-*negative constant* *c* *satisfying* $0\leq c<\frac{1}{n}\sum_{i=1}^{n}x_{i}$, *one has*

9 $$ \biggl( \frac{x_{1}}{\sum_{i=1}^{n}x_{i}},\ldots,\frac{x_{n}}{\sum_{i=1} ^{n}x_{i}} \biggr) \prec \biggl( \frac{x_{1}-c}{\sum_{i=1}^{n}(x_{i}-c)},\ldots,\frac{x_{n}-c}{\sum_{i=1}^{n}(x_{i}-c)} \biggr) . $$

## Proof of main result

### Proof of Theorem 1

Note that the generalized geometric Bonferroni mean is defined by

$$ \operatorname {GB}^{p,q,r}(\boldsymbol {x})=\frac{1}{p+q+r}\prod^{n}_{i,j,k=1,i\neq j\neq k}(p x_{i}+q x_{j}+r x_{k})^{\frac{1}{n(n-1)(n-2)}}, $$

taking the natural logarithm gives

$$ \log \operatorname {GB}^{p,q,r}(\boldsymbol {x}) = \log \frac{1}{p+q+r}+\frac{1}{n(n-1)(n-2)}Q, $$

where

$$\begin{aligned} Q &= \sum^{n}_{j,k=3,j \neq k} \bigl[ \log (p x_{1}+q x_{j}+r x_{k})+ \log (p x_{2}+q x_{j}+r x_{k}) \bigr] \\ &\quad{} +\sum^{n}_{i,k=3,i \neq k} \bigl[ \log (p x_{i}+q x_{1}+r x_{k})+ \log (p x_{i}+q x_{2}+r x_{k}) \bigr] \\ &\quad{}+\sum^{n}_{i,j=3,i \neq j} \bigl[ \log (p x_{i}+q x_{j}+r x_{1})+ \log (p x_{i}+q x_{j}+r x_{2}) \bigr] \\ &\quad{}+\sum^{n}_{k=3} \bigl[\log (px_{1}+qx_{2}+ r x_{k})+\log (px_{2}+q x_{1}+ r x_{k}) \bigr] \\ &\quad{}+\sum^{n}_{j=3} \bigl[\log (px_{1}+qx_{j}+ r x_{2})+\log (px_{2}+q x_{j}+ r x_{1}) \bigr] \\ &\quad{}+\sum^{n}_{i=3} \bigl[\log (px_{i}+qx_{1}+ r x_{2})+\log (px_{i}+q x_{2}+ r x_{1}) \bigr] \\ &\quad{}+\sum^{n}_{i,j,k=3, i\neq j\neq k}\log (p x_{i}+q x_{j}+rx_{k}). \end{aligned}$$

Differentiating $\operatorname {GB}^{p,q,r}(\boldsymbol {x})$ with respect to $x_{1}$ and $x_{2}$, respectively, we have

$$\begin{aligned}& \begin{aligned} \frac{\partial \operatorname {GB}^{p,q,r}(\boldsymbol {x})}{\partial x_{1}} &= \frac{\operatorname {GB}^{p,q,r}(\boldsymbol {x})}{n(n-1)(n-2)}\cdot \frac{\partial Q}{\partial x_{1}} \\ &=\frac{\operatorname {GB}^{p,q,r}(\boldsymbol {x})}{n(n-1)(n-2)} \Biggl[\sum^{n}_{j,k=3,j \neq k} \frac{p}{p x_{1}+q x_{j}+r x_{k}} +\sum^{n}_{i,k=3,i \neq k} \frac{q}{p x_{i}+q x_{1}+r x_{k}} \\ &\quad{}+\sum^{n}_{i,j=3,i \neq j}\frac{r}{p x_{i}+q x_{j}+r x_{1}} + \sum^{n}_{k=3} \biggl(\frac{p}{px_{1}+qx_{2}+ r x_{k}}+ \frac{q}{px_{2}+q x_{1}+ r x_{k}} \biggr) \\ &\quad{}+\sum^{n}_{j=3} \biggl( \frac{p}{px_{1}+qx_{j}+ r x_{2}}+ \frac{r}{px_{2}+q x _{j}+ r x_{1}} \biggr) \\ &\quad{} +\sum^{n}_{i=3} \biggl( \frac{q}{px_{i}+qx_{1}+ r x_{2}}+\frac{r}{px _{i}+q x_{2}+ r x_{1}} \biggr) \Biggr], \end{aligned} \\ & \begin{aligned} \frac{\partial \operatorname {GB}^{p,q,r}(\boldsymbol {x})}{\partial x_{2}}&= \frac{\operatorname {GB}^{p,q,r}(\boldsymbol {x})}{n(n-1)(n-2)}\cdot \frac{\partial Q}{\partial x_{2}} \\ &=\frac{\operatorname {GB}^{p,q,r}(\boldsymbol {x})}{n(n-1)(n-2)} \Biggl[\sum^{n}_{j,k=3,j \neq k} \frac{p}{p x_{2}+q x_{j}+r x_{k}} +\sum^{n}_{i,k=3,i \neq k} \frac{q}{p x_{i}+q x_{2}+r x_{k}} \\ &\quad{} +\sum^{n}_{i,j=3,i \neq j}\frac{r}{p x_{i}+q x_{j}+r x_{2}} +\sum^{n}_{k=3} \biggl(\frac{q}{px_{1}+qx_{2}+ r x_{k}}+ \frac{p}{px_{2}+q x_{1}+ r x_{k}} \biggr) \\ &\quad{} +\sum^{n}_{j=3} \biggl( \frac{r}{px_{1}+qx_{j}+ r x_{2}}+ \frac{p}{px_{2}+q x _{j}+ r x_{1}} \biggr) \\ &\quad{} +\sum^{n}_{i=3} \biggl( \frac{r}{px_{i}+qx_{1}+ r x_{2}}+\frac{q}{px _{i}+q x_{2}+ r x_{1}} \biggr) \Biggr]. \end{aligned} \end{aligned}$$

It is easy to see that $\operatorname {GB}^{p,q,r}(\boldsymbol {x})$ is symmetric on $\mathbb{R}^{n}_{++}$. For $n\geq 3 $, we have

$$\begin{aligned} \Delta_{1}: ={}&(x_{1} -x_{2}) \biggl[ \frac{\partial \operatorname {GB}^{p,q,r}(\boldsymbol {x})}{ \partial x_{1}}-\frac{\partial \operatorname {GB}^{p,q,r}(\boldsymbol {x})}{\partial x_{2}} \biggr] \\ ={} &\frac{(x_{1} -x_{2})\operatorname {GB}^{p,q,r}(\boldsymbol {x})}{n(n-1)(n-2)} \Biggl[p \sum^{n}_{j,k=3,j \neq k} \biggl(\frac{1}{p x_{1}+q x_{j}+r x_{k}}-\frac{1}{p x _{2}+q x_{j}+r x_{k}} \biggr) \\ &{} +q\sum^{n}_{i,k=3,i \neq k} \biggl( \frac{1}{p x_{i}+q x_{1}+r x_{k}}- \frac{1}{p x_{i}+q x_{2}+r x_{k}} \biggr) \\ &{}+r\sum^{n}_{i,j=3,i \neq j} \biggl( \frac{1}{p x_{i}+q x_{j}+r x_{1}}- \frac{1}{p x_{i}+q x_{j}+r x_{2}} \biggr) \\ &{}+\sum^{n}_{k=3} \biggl( \frac{p-q}{px_{1}+qx_{2}+ r x_{k}}+ \frac{q-p}{px _{2}+q x_{1}+ r x_{k}} \biggr) \\ &{}+\sum^{n}_{j=3} \biggl( \frac{p-r}{px_{1}+qx_{j}+ r x_{2}}+ \frac{r-p}{px _{2}+q x_{j}+ r x_{1}} \biggr) \\ &{}+\sum^{n}_{i=3} \biggl( \frac{q-r}{px_{i}+qx_{1}+ r x_{2}}+ \frac{r-q}{px _{i}+q x_{2}+ r x_{1}} \biggr) \Biggr] \\ ={}&-\frac{(x_{1} -x_{2})^{2}\operatorname {GB}^{p,q,r}(\boldsymbol {x})}{n(n-1)(n-2)} \Biggl[ \sum^{n}_{j,k=3,j \neq k} \frac{p^{2}}{(p x_{1}+q x_{j}+r x_{k})(p x _{2}+q x_{j}+r x_{k})} \\ &{}+\sum^{n}_{i,k=3,i \neq k}\frac{q^{2}}{(p x_{i}+q x_{1}+r x_{k})(p x _{i}+q x_{2}+r x_{k})} \\ &{}+\sum^{n}_{i,j=3,i \neq j}\frac{r^{2}}{(p x_{i}+q x_{j}+r x_{1})(p x _{i}+q x_{j}+r x_{2})} \\ &{}+\sum^{n}_{k=3}\frac{(p-q)^{2}}{(px_{1}+qx_{2}+ r x_{k})(px_{2}+q x _{1}+ r x_{k})} \\ &{}+\sum^{n}_{j=3}\frac{(p-r)^{2}}{(px_{1}+qx_{j}+ r x_{2})(px_{2}+q x _{j}+ r x_{1})} \\ &{}+\sum^{n}_{i=3}\frac{(q-r)^{2}}{(px_{i}+qx_{1}+ r x_{2})(px_{i}+q x _{2}+ r x_{1})} \Biggr]. \end{aligned}$$

This implies that $\Delta_{1}\leq 0$ for $\boldsymbol {x}\in \mathbb{R}^{n} _{++}$ ($n\geq 3$). By Proposition 2, we conclude that $\operatorname {GB}^{p,q,r}(\boldsymbol {x})$ is Schur concave on $\mathbb{R}^{n}_{++}$.

In view of the discrimination criterion of Schur geometrically convexity, we start with the following calculations:

$$\begin{aligned} \Delta_{2}: ={}&(\log x_{1} - \log x_{2}) \biggl[ x_{1}\frac{\partial \operatorname {GB}^{p,q,r}(\boldsymbol {x})}{\partial x_{1}}-x_{2}\frac{\partial \operatorname {GB}^{p,q,r}(\boldsymbol {x})}{ \partial x_{2}} \biggr] \\ = {}&\frac{(\log x_{1} - \log x_{2})\operatorname {GB}^{p,q,r}(\boldsymbol {x})}{n(n-1)(n-2)} \\ &{}\times \Biggl[p \sum^{n}_{j,k=3,j \neq k} \biggl( \frac{x_{1}}{p x_{1}+q x _{j}+r x_{k}}-\frac{x_{2}}{p x_{2}+q x_{j}+r x_{k}} \biggr) \\ &{}+q\sum^{n}_{i,k=3,i \neq k} \biggl( \frac{x_{1}}{p x_{i}+q x_{1}+r x_{k}}- \frac{x _{2}}{p x_{i}+q x_{2}+r x_{k}} \biggr) \\ &{}+r\sum^{n}_{i,j=3,i \neq j} \biggl( \frac{x_{1}}{p x_{i}+q x_{j}+r x_{1}}- \frac{x _{2}}{p x_{i}+q x_{j}+r x_{2}} \biggr) \\ &{}+\sum^{n}_{k=3} \biggl( \frac{px_{1}-qx_{2}}{px_{1}+qx_{2}+ r x_{k}}+ \frac{qx _{1}-px_{2}}{px_{2}+q x_{1}+ r x_{k}} \biggr) \\ &{}+\sum^{n}_{j=3} \biggl( \frac{px_{1}-rx_{2}}{px_{1}+qx_{j}+ r x_{2}}+ \frac{rx _{1}-px_{2}}{px_{2}+q x_{j}+ r x_{1}} \biggr) \\ &{}+\sum^{n}_{i=3} \biggl( \frac{qx_{1}-rx_{2}}{px_{i}+qx_{1}+ r x_{2}}+ \frac{rx _{1}-qx_{2}}{px_{i}+q x_{2}+ r x_{1}} \biggr) \Biggr] \\ ={} &\frac{(x_{1} -x_{2})(\log x_{1} - \log x_{2})\operatorname {GB}^{p,q,r}(\boldsymbol {x})}{n(n-1)(n-2)} \\ &{}\times \Biggl[\sum^{n}_{j,k=3,j \neq k} \frac{q x_{j}+r x_{k}}{(p x _{1}+q x_{j}+r x_{k})(p x_{2}+q x_{j}+r x_{k})} \\ &{}+\sum^{n}_{i,k=3,i \neq k}\frac{px_{i}+r x_{k}}{(p x_{i}+q x_{1}+r x _{k})(p x_{i}+q x_{2}+r x_{k})} \\ &{}+\sum^{n}_{i,j=3,i \neq j}\frac{px_{i}+q x_{j}}{(p x_{i}+q x_{j}+r x _{1})(p x_{i}+q x_{j}+r x_{2})} \\ &{}+\sum^{n}_{k=3}\frac{2pq(x_{1}+x_{2})+rx_{k}(p+q)}{(px_{1}+qx_{2}+ r x_{k})(px_{2}+q x_{1}+ r x_{k})} \\ &{}+\sum^{n}_{j=3}\frac{2rp(x_{1}+x_{2})+qx_{j}(p+r)}{(px_{1}+qx_{j}+ r x_{2})(px_{2}+q x_{j}+ r x_{1})} \\ &{}+\sum^{n}_{i=3}\frac{2qr(x_{1}+x_{2})+px_{i}(q+r)}{(px_{i}+qx_{1}+ r x_{2})(px_{i}+q x_{2}+ r x_{1})} \Biggr]. \end{aligned}$$

Thus, we have $\Delta_{2} \geq 0$ for $\boldsymbol {x}\in \mathbb{R}^{n}_{++} $ ($n\geq 3$). It follows from Proposition 3 that $\operatorname {GB}^{p,q,r}(\boldsymbol {x})$ is Schur geometric convex on $\mathbb{R}^{n}_{++}$.

Finally, we discuss the Schur harmonic convexity of $\operatorname {GB}^{p,q,r}(\boldsymbol {x})$. A direct computation gives

$$\begin{aligned} \Delta_{3}: ={}&(x_{1} -x_{2}) \biggl( x^{2}_{1}\frac{\partial \operatorname {GB}^{p,q,r}(\boldsymbol {x})}{ \partial x_{1}}-x^{2}_{2} \frac{\partial \operatorname {GB}^{p,q,r}(\boldsymbol {x})}{\partial x_{2}} \biggr) \\ = {}&\frac{(x_{1} -x_{2})\operatorname {GB}^{p,q,r}(\boldsymbol {x})}{n(n-1)(n-2)} \Biggl[p \sum^{n}_{j,k=3,j \neq k} \biggl(\frac{x^{2}_{1}}{p x_{1}+q x_{j}+r x_{k}}-\frac{x ^{2}_{2}}{p x_{2}+q x_{j}+r x_{k}} \biggr) \\ &{}+q\sum^{n}_{i,k=3,i \neq k} \biggl( \frac{x^{2}_{1}}{p x_{i}+q x_{1}+r x _{k}}- \frac{x^{2}_{2}}{p x_{i}+q x_{2}+r x_{k}} \biggr) \\ &{}+r\sum^{n}_{i,j=3,i \neq j} \biggl( \frac{x^{2}_{1}}{p x_{i}+q x_{j}+r x _{1}}- \frac{x^{2}_{2}}{p x_{i}+q x_{j}+r x_{2}} \biggr) \\ &{}+\sum^{n}_{k=3} \biggl( \frac{px^{2}_{1}-qx^{2}_{2}}{px_{1}+qx_{2}+ r x_{k}}+ \frac{qx^{2}_{1}-px ^{2}_{2}}{px_{2}+q x_{1}+ r x_{k}} \biggr) \\ &{}+\sum^{n}_{j=3} \biggl( \frac{px^{2}_{1}-rx^{2}_{2}}{px_{1}+qx_{j}+ r x_{2}}+\frac{rx^{2}_{1}-px ^{2}_{2}}{px_{2}+q x_{j}+ r x_{1}} \biggr) \\ &{}+\sum^{n}_{i=3} \biggl( \frac{qx^{2}_{1}-rx^{2}_{2}}{px_{i}+qx_{1}+ r x_{2}}+\frac{rx^{2}_{1}-qx ^{2}_{2}}{px_{i}+q x_{2}+ r x_{1}} \biggr) \Biggr] \\ ={}&\frac{(x_{1} -x_{2})^{2}\operatorname {GB}^{p,q,r}(\boldsymbol {x})}{n(n-1)(n-2)} \Biggl[\sum^{n}_{j,k=3,j \neq k} \frac{(x_{1}+x_{2})(q x_{j}+r x_{k})+px_{1}x_{2}}{(p x_{1}+q x_{j}+r x_{k})(p x_{2}+q x_{j}+r x_{k})} \\ &{}+\sum^{n}_{i,k=3,i \neq k}\frac{(x_{1}+x_{2})(px_{i}+r x_{k})+qx _{1}x_{2}}{(p x_{i}+q x_{1}+r x_{k})(p x_{i}+q x_{2}+r x_{k})} \\ &{}+\sum^{n}_{i,j=3,i \neq j}\frac{(x_{1}+x_{2})(px_{i}+q x_{j})+rx _{1}x_{2}}{(p x_{i}+q x_{j}+r x_{1})(p x_{i}+q x_{j}+r x_{2})} \\ &{}+\sum^{n}_{k=3}\frac{2pq(x^{2}_{1}+x^{2}_{2})+rx_{k}(x_{1}+x_{2})(p+q)+x _{1}x_{2}(p+q)^{2}}{(px_{1}+qx_{2}+ r x_{k})(px_{2}+q x_{1}+ r x_{k})} \\ &{}+\sum^{n}_{j=3}\frac{2pr(x^{2}_{1}+x^{2}_{2})+qx_{j}(x_{1}+x_{2})(p+r)+x _{1}x_{2}(p+r)^{2}}{(px_{1}+qx_{j}+ r x_{2})(px_{2}+q x_{j}+ r x_{1})} \\ &{}+\sum^{n}_{i=3}\frac{2qr(x^{2}_{1}+x^{2}_{2})+px_{i}(x_{1}+x_{2})(q+r)+x _{1}x_{2}(q+r)^{2}}{(px_{i}+qx_{1}+ r x_{2})(px_{i}+q x_{2}+ r x_{1})} \Biggr]. \end{aligned}$$

Hence, we obtain $\Delta_{3}\geq 0$ for $\boldsymbol {x}\in \mathbb{R}^{n} _{++}$ ($n\geq 3$). Using Proposition 4 leads to the assertion that $\operatorname {GB}^{p,q,r}(\boldsymbol {x})$ is Schur harmonic convex on $\mathbb{R}^{n}_{++}$.

The proof of Theorem 1 is completed. □

### Remark 5

As a direct consequence of Theorem 1, taking $r=0$ in Theorem 1 together with the identity $\operatorname {GB}^{p,q,0}(\boldsymbol {x})=\operatorname {GB}^{p,q}(\boldsymbol {x})$, we arrive at the assertion of Corollary 1.

## Applications

As an application of Theorem 1, we establish the following interesting inequalities for generalized geometric Bonferroni mean.

### Theorem 2

*Let* *p*, *q*, *r* *be non*-*negative real numbers with* $p+q+r \neq 0$. *Then*, *for arbitrary* $\boldsymbol {x}\in \mathbb{R}^{n}_{++}$ ($n\geq 3 $),

10 $$ \operatorname {GB}^{p,q,r}(\boldsymbol {x}) \leq A_{n}(\boldsymbol {x}). $$

### Proof

It follows from Theorem 1 that $\operatorname {GB}^{p,q,r}(\boldsymbol {x})$ is Schur concave on $\mathbb{R}^{n}_{++}$.

Using Lemma 1, one has

$$ \bigl( \underbrace{ A_{n}(\boldsymbol {x}), A_{n}(\boldsymbol {x}),\ldots, A_{n}(\boldsymbol {x})}_{n} \bigr) \prec (x_{1}, x_{2},\ldots, x_{n}). $$

Thus, we deduce from Definition 1 that

$$ \operatorname {GB}^{p,q,r} \bigl( A_{n}(\boldsymbol {x}), A_{n}(\boldsymbol {x}), \ldots, A_{n}(\boldsymbol {x}) \bigr) \geq \operatorname {GB}^{p,q,r}(x_{1}, x_{2},\ldots, x_{n}), $$

which implies that

$$ A_{n}(\boldsymbol {x}) \geq \operatorname {GB}^{p,q,r}(\boldsymbol {x}). $$

Theorem 2 is proved. □

### Theorem 3

*Let* *p*, *q*, *r* *be non*-*negative real numbers with* $p+q+r \neq 0$, *and let* *c* *be a constant satisfying* $0\leq c< A_{n}(\boldsymbol {x})$, $(\boldsymbol {x}-c)=(x_{1}-c,x_{2}-c,\ldots,x_{n}-c)$. *Then*, *for arbitrary* $\boldsymbol {x}\in \mathbb{R}^{n}_{++}$ ($n\geq 3$),

11 $$ \operatorname {GB}^{p,q,r }(\boldsymbol {x}-c)\leq \biggl( 1-\frac{c}{A_{n}(\boldsymbol {x})} \biggr) \operatorname {GB}^{p,q,r}(\boldsymbol {x}). $$

### Proof

By the majorization relationship given in Lemma 2,

$$ \biggl( \frac{x_{1}}{\sum_{i=1}^{n}x_{i}},\ldots,\frac{x_{n}}{\sum_{i=1} ^{n}x_{i}} \biggr) \prec \biggl( \frac{x_{1}-c}{\sum_{i=1}^{n}(x_{i}-c)},\ldots,\frac{x_{n}-c}{\sum_{i=1}^{n}(x_{i}-c)} \biggr) , $$

it follows from Theorem 1 that

$$ \operatorname {GB}^{p,q,r} \biggl( \frac{x_{1}}{\sum_{i=1}^{n}x_{i}},\ldots,\frac{x_{n}}{ \sum_{i=1}^{n}x_{i}} \biggr) \geq \operatorname {GB}^{p,q,r} \biggl( \frac{x_{1}-c}{\sum_{i=1}^{n}(x_{i}-c)},\ldots,\frac{x_{n}-c}{\sum_{i=1}^{n}(x_{i}-c)} \biggr) , $$

that is,

$$ \frac{\operatorname {GB}^{p,q,r}(x_{1},x_{2},\ldots,x_{n})}{\sum_{i=1}^{n}x_{i}} \geq \frac{\operatorname {GB}^{p,q,r}(x_{1}-c,x_{2}-c,\ldots,x_{n}-c)}{\sum_{i=1} ^{n}(x_{i}-c)}, $$

which implies that

$$ \operatorname {GB}^{p,q,r }(\boldsymbol {x}-c)\leq \biggl( 1-\frac{c}{A_{n}(\boldsymbol {x})} \biggr) \operatorname {GB}^{p,q,r}(\boldsymbol {x}). $$

This completes the proof of Theorem 3. □

## Conclusion

This paper is a follow-up study of our recent work [24], we generalize the geometric Bonferroni mean by introducing three non-negative parameters *p*, *q*, *r*, under the condition of $p+q+r\neq 0$, we prove that the generalized geometric Bonferroni mean $\operatorname {GB}^{p,q,r}(\boldsymbol {x})$ is Schur concave, Schur geometric convex and Schur harmonic convex on $\mathbb{R}^{n}_{++}$. As an application of the Schur convexity, we establish two inequalities for generalized geometric Bonferroni mean. In fact, there have been a large number inequalities for means which originate from the Schur convexity of functions. For details, we refer the interested reader to [27–32] and the references therein.
